# Drought and Nitrogen Addition Modulate Wolf Spider‐Associated Responses in a Detrital Food Web Without Detectable Functional Cascades

**DOI:** 10.1002/ece3.74165

**Published:** 2026-08-09

**Authors:** Bingchuan Zhang, Zhiwei Zhang, Wang Ma, Zhengwen Wang

**Affiliations:** ^1^ Erguna Forest‐Steppe Ecotone Research Station, CAS Key Laboratory of Forest Ecology and Silviculture, Institute of Applied Ecology Chinese Academy of Sciences Shenyang China; ^2^ School of Life Sciences Liaocheng University Liaocheng China

**Keywords:** detrital food webs, drought, grassland, nitrogen deposition, trophic cascade effects, wolf spider

## Abstract

Predators play a key role in sustaining biodiversity and ecological functioning in grasslands, yet their ecological roles may shift under increasingly severe droughts and nitrogen (N) deposition. However, how top‐down effects of predators on detrital food webs respond to these global change factors remains unclear. Here, we conducted a field mesocosm experiment in a temperate grassland where we manipulated the presence/absence of locally dominant ground‐dwelling wolf spiders (Lycosidae) under drought and N addition. We examined how these predators influence soil fauna and microbial communities, and how such changes cascade to soil N availability and litter decomposition. Drought and N addition independently modulated wolf spider‐associated effects on the detrital food web by altering soil fauna and microbial community responses. However, these community‐level changes did not cascade to detectable shifts in soil inorganic N availability or litter decomposition rates. Drought altered spider‐induced changes in fungal and bacterial diversity and also altered spider effects on the community composition of Collembola, fungi, and bacteria. In contrast, N addition mainly increased the sensitivity of Collembola abundance to spider presence, while having little effect on the microbial communities. Overall, our findings highlight that drought and N addition can modulate detrital food webs and predator–prey interactions, providing new insight into how global change may alter predator‐associated effects in detrital food webs. These findings suggest that functional redundancy within detritivore and microbial assemblages may help buffer soil ecosystem functioning under global change, highlighting the importance of conserving biodiversity even if it is in a state of redundancy.

## Graphical abstract

Predators are important regulators of grassland biodiversity and ecosystem functioning, but their top‐down effects on detrital food webs under global change remain poorly understood. Using a field mesocosm experiment in a temperate grassland, we show that drought and nitrogen addition independently altered the effects of wolf spiders on soil fauna and microbial communities, but not on soil inorganic nitrogen or litter decomposition.

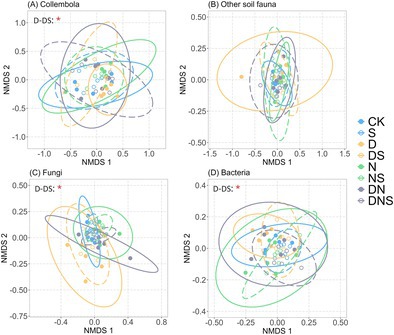

## 1 Introduction

Trophic cascades are key processes regulating population dynamics, community structure, biodiversity, and ecosystem functions in grassland ecosystems (Schmitz 2007; Letourneau et al. 2009; Liu et al. 2016). However, global change is reshaping ecological interactions (Valiente‐Banuet et al. 2015; Yang et al. 2021). In particular, frequent drought events and increased N deposition are leading to declines in biodiversity and mismatches in interactions between species, especially those at adjacent trophic levels (Bobbink et al. 2010; Poisot et al. 2015; Ault 2020). The responses of individual predators or predator populations to these global change factors can either amplify or dampen their trophic cascading effects (Lensing and Wise 2006; Liu et al. 2018; Hu et al. 2024). Predators at higher trophic levels may respond directly to environmental changes, altering prey community composition and thereby modifying ecosystem functions (Koltz et al. 2018). Such cross‐trophic covariation strongly influences key ecological processes, including productivity, biodiversity maintenance, and litter decomposition (Schmitz 2006; Liu et al. 2016; Jia et al. 2018). Therefore, linking the responses of populations or communities across trophic levels can provide a key framework for predicting how global change affects ecosystem functioning.

While many trophic‐cascade studies have focused on predator effects on aboveground herbivores and plant production, predators may also regulate belowground detrital pathways by altering detritivore communities, microbial dynamics, and decomposition processes. Spiders, among the most abundant and diverse predators in terrestrial ecosystems, can shape belowground detrital food webs through their effects on detritivore and microbial communities. Mounting evidence suggests that the top‐down effects of spiders on detrital food webs are strongly influenced by global change (Liu et al. 2018; Koltz et al. 2018). Given their key trophic position, ecologists have increasingly focused on the cascading effects of spiders on ecosystem functions such as litter decomposition and soil N availability (Schmitz 2008, 2009; Kajak 1997; Koltz et al. 2018). Previous studies have shown that drought and climate warming can reverse the effects of spider density on litter decomposition rates in tundra and forest ecosystems (Koltz et al. 2018; Liu et al. 2015). However, Lensing and Wise (2006) found that the cascading effects of spiders on litter decomposition were modulated by rainfall, with spiders accelerating decomposition under low but not high rainfall, indicating strong hydrological control over predator effects. In addition, N addition can directly reduce Collembola density, thereby amplifying the negative effects of spiders on litter decomposition (Liu et al. 2018). In summary, the trophic cascading effects of spiders within detrital food webs vary with environmental context under global change. This variation highlights the importance of understanding how environmental change influences prey communities, microbial dynamics, and soil resource conditions that may mediate spider‐associated effects on ecosystem functioning.

Previous studies have shown that spider‐mediated cascading effects can influence soil N availability and litter decomposition by regulating soil fauna and microbial communities within detrital food webs (Schmitz 2008; Liu et al. 2016; Koltz et al. 2018). As one of the primary prey groups of spiders, Collembola directly affect microbial biomass and activity through their feeding on fungi and bacteria, and indirectly influence microbial processes by fragmenting organic matter and modifying nutrient availability (Caravaca and Ruess 2014; Anslan et al. 2018; Coulibaly et al. 2019; Lucas et al. 2020). Microorganisms play central roles in soil ecosystem functioning, directly mediating key processes such as nutrient cycling and litter decomposition (Fierer et al. 2013; Glassman et al. 2018). As primary decomposers in detrital food webs, fungi and bacteria are essential for maintaining food web stability and serving as an important energy base for meso‐ and microfauna. Collectively, these links may be potential pathways through which spider‐mediated top‐down cascades indirectly shape ecosystem functioning.

Despite growing recognition of spider‐mediated cascades and their mechanistic links to soil biota, empirical evidence remains highly context dependent (Koltz et al. 2018; Lensing and Wise 2006; Liu et al. 2018). The direction and strength of these cascades vary considerably across ecosystems and environmental conditions, suggesting that their variation may be shaped by both biotic composition and abiotic constraints. Therefore, understanding when and where spiders exert strong top‐down control on detrital processes requires system‐specific experiments that integrate multiple global change factors. Grassland ecosystems, in particular, provide an ideal context to test these dynamics due to their high sensitivity to drought and nutrient enrichment. Based on our long‐term field surveys, wolf spiders (Lycosidae) are among the dominant surface‐active invertebrate predators in the Hulunbuir grasslands and remain active throughout the growing season. Wolf spiders inhabit nearly all terrestrial habitats and are well known for mediating cascading effects on detrital food webs and decomposition processes across diverse ecosystems (Maran and Pelini 2016; Wise et al. 1999). Their widespread distribution and key ecological role within detrital food webs make them suitable model predators for investigating how multi‐trophic interactions mediate ecosystem responses to global change.

In this study, we conducted a mesocosm experiment in a temperate grassland in northern China, manipulating spider presence/absence under drought, N addition, and their combination. Our objectives were to determine how drought and N addition influence the wolf spider‐associated effects within detrital food webs, soil nutrient availability, and litter decomposition rates. Specifically, we addressed the following questions: (i) Does wolf spider presence alter soil fauna and microbial communities, and are these community‐level changes associated with soil N availability and litter decomposition? (ii) How do drought and N addition modulate the strength and direction of wolf spider‐induced trophic cascades through soil fauna and microbial communities, and do these changes ultimately affect soil N availability and litter decomposition?

## 2 Materials and Methods

### 2.1 Study Site & Experimental Design

This study was conducted at the Erguna Forest‐Steppe Ecotone Research Station, Institute of Applied Ecology, Chinese Academy of Sciences, from 2020 to 2021. The experiment was carried out in a natural temperate grassland site (N50°10′46″, E119°22′56″) that has been fenced since 2013. The mean annual temperature is −2°C, and the mean annual precipitation is 363 mm, about 75% of which falls during the growing season. Plant communities are dominated by *Leymus chinensis*, *Stipa baicalensis*, *Artemisia frigida* , *Thermopsis lanceolata*, *Cymbaria dahurica*, and *Carex duriuscula* . According to the U.S. soil classification standard, the soil is classified as chernozem.

To examine the effects of wolf spiders on soil fauna, microbial communities, soil N availability and litter decomposition under ambient conditions, drought, N addition, and their combination, we employed a full‐factorial field experiment. The drought treatment was simulated by intercepting 66% of the natural rainfall from May to August using rainout shelters with evenly spaced, highly light‐transmissive polycarbonate strips that covered 2/3 of the vertical projected roof area. Watertight aluminum plates were vertically inserted around the drought plots to a depth of 1 m below the soil surface to prevent external subsurface water from permeating into the drought plots. Supported by arched stainless‐steel frames, the shelter roofs were suspended 2.5 and 1 m above the ground at the highest and lowest points, respectively, creating a sloped roof surface that facilitated runoff of intercepted rainfall. This design allowed air flow below the panels to minimize potential greenhouse effects. This rainout shelter design has been used in a wide range of ecosystems globally due to its low cost and minimal impact on the microclimate (Knapp et al. 2024). Soil moisture under ambient and drought conditions during the spider experiment is shown in Figure S1. Nitrogen addition was implemented in mid‐May by spraying dissolved NH_4_NO_3_ evenly across designated plots at a rate of 10 g N m^−2^ year^−1^, while non‐N plots received an equivalent volume of purified water to control for water addition. Drought (66% natural precipitation reduction), N addition (10 g NH_4_NO_3_ m^−2^ year^−1^), and their combination have been implemented annually every spring since 2018 in a randomized block design with six replicates per treatment. Each 6 m × 6 m plot was separated by a 2 m buffer strip. In 2021, based on this experiment, we added a mesocosm manipulation to assess the top‐down effects of wolf spiders. The experiment followed a randomized block design with six blocks, each containing four environmental treatments: ambient control, drought, N addition, and drought plus N addition. Within each 6 m × 6 m plot, we established a pair of mesocosms, one with two female wolf spiders inside and the other one without wolf spiders.

We installed paired mesocosms in each plot from May to August, resulting in 48 mesocosms across 24 plots. Each mesocosm was 75 cm × 75 cm × 75 cm in size and was supported by a steel frame covered with 1 mm fiberglass mesh to prevent spiders entering or escaping, and to avoid predators such as birds, rodents or frogs. The lower part of each steel frame was buried 15 cm into the soil to minimize potential exchange of soil fauna between paired mesocosms. Mesocosms within each plot were positioned at least 1 m apart to avoid shading effects and to further reduce potential movement of soil fauna between mesocosms. To explore the top‐down cascading effects of wolf spiders, we added two locally collected wolf spiders to one mesocosm of each pair on May 20 and maintained the spider treatment until August 20, enabling direct comparison between spider‐present and spider‐absent conditions during the growing season. This corresponded to a density of 3.56 individuals m^−2^, which was higher than the mean field density observed at our site (1.48 ± 1.1 individuals m^−2^), but lower than the maximum observed field density during this period (8 individuals m^−2^). This modestly elevated density was used to establish a clear predator‐presence treatment while maintaining ecological relevance, and the same density was applied across all drought and N addition treatments to allow standardized comparisons. Before starting the experiment, other predatory arthropods were excluded from all the mesocosms. In total, 48 female wolf spiders (6 blocks × 4 treatments per block × 2 spiders per plot) were released into 24 mesocosms.

### 2.2 Litter Collection & Installation

Two litterbags (20 cm × 10 cm, 1 mm mesh) were placed on the soil surface inside each mesocosm in late September 2020 to estimate the litter decomposition rates during spider treatments. Each litterbag was filled with 5.0 g of air‐dried leaf litter from the dominant species *L. chinensis* , which was collected from adjacent undisturbed grassland. Litterbags were retrieved from all mesocosms in May and August 2021 and transported to the laboratory. In the lab, the litterbags were dried at 70°C to a constant mass after carefully removing attached soil particles and any soil fauna that had entered the bags during field exposure.

### 2.3 Soil Fauna Sampling & Classification

After litterbag collection, soil fauna from the litter layer and the soil surface near each retrieved litterbag were collected using a suction sampler, targeting primarily surface‐ and litter‐dwelling arthropods. All the collected soil fauna were preserved in 95% ethyl alcohol and identified to the finest possible taxonomic level (Yin 2000). The identified soil fauna were classified into two groups, Collembola (springtails) and other soil fauna, and the individuals in each group were counted. As potential prey of spiders (Nentwig 1987), the Collembola communities mainly consisted of the following families: Entomobryidae, Isotomidae, and Onychiuridae. Other soil fauna included Diptera larvae, Psocoptera, Coleoptera, and Hymenoptera.

### 2.4 Soil & Microbe Sampling

After litterbag retrieval and soil fauna sampling, soil samples were collected from the 0–10 cm layer at three random locations beneath the litterbags in each mesocosm using a 7.5 cm diameter soil corer. The three samples per mesocosm were combined and passed through a 2 mm sieve. Each mixed soil sample was divided into subsamples for the following analyses: (i) Soil nutrients: soil N availability (the sum of soil inorganic N: NO_3_^−^ and NH_4_^+^) was extracted with 2 M KCl and quantified using an automatic flow analyzer (AA3, Bran + Luebbe, Germany); (ii) microbial biomass: Microbial biomass carbon (MBC) and nitrogen (MBN) were measured using the CHCl_3_ fumigation‐extraction procedure (Brookes et al. 1985); (iii) microbial diversity: Microbial community composition and diversity were analyzed using amplicon sequencing of bacterial 16S rRNA genes and fungal ITS regions. Total genomic DNA was first extracted from each sample, and PCR was then used to amplify the target marker regions. For bacterial community analysis, the V4 region of the 16S rRNA gene was amplified using primers 515F and 806R. For fungal community analysis, the ITS1 region was amplified using primers ITS5‐1737F and ITS2‐2043R. PCR products were checked by 2% agarose gel electrophoresis, purified, quantified, pooled in equimolar amounts, and used for library construction. Sequencing was performed on an Illumina NovaSeq 6000 platform to generate 250 bp paired‐end reads. Raw paired‐end reads were assigned to samples based on their barcodes, and barcode and primer sequences were removed. Paired‐end reads were merged using FLASH v1.2.7. Raw tags were quality‐filtered using the QIIME v1.9.1 pipeline, and chimeric sequences were removed using VSEARCH to obtain effective tags. Effective tags were clustered into operational taxonomic units (OTUs) at 97% sequence similarity using UPARSE v7.0.1001. The most abundant sequence within each OTU was selected as the representative sequence for taxonomic assignment. For bacterial communities, representative sequences were taxonomically annotated using the Mothur method against the SILVA database to obtain bacterial taxonomic information. For fungal communities, representative sequences were assigned using the BLAST method in QIIME against the UNITE database to obtain fungal taxonomic information. The resulting bacterial and fungal OTU abundance tables were used for subsequent analyses.

### 2.5 Statistical Analyses

Linear mixed‐effects models (LMMs) were used to evaluate how drought and N addition influenced spider‐mediated regulation in detrital food webs. For each response variable, spider‐mediated regulation was calculated as the within‐pair difference between spider‐present and spider‐absent mesocosms within the same plot. Specifically, we analyzed these spider‐mediated responses for Collembola abundance and diversity, other soil fauna abundance and diversity, MBC, MBN, fungal and bacterial diversity, soil N availability, and litter decomposition rate. Soil N availability was treated as a state variable reflecting the outcome of multiple N cycling processes, whereas litter decomposition rate was used as an indicator of ecosystem functioning. Drought, N addition, and their interaction were included as fixed effects, and block was included as a random effect. This model was specified as: Spider‐mediated response ~ Drought * N addition + (1|Block). To facilitate comparison across variables measured on different scales, spider‐mediated responses were min–max normalized to a 0–1 range. These normalized values should be interpreted as relative effect magnitudes rather than absolute effect sizes in the original units. In addition, we fitted LMM using the mesocosm‐level data to test the direct and interactive effects of drought, N addition, and spider presence. In these models, drought, N addition, spider presence, and their interactions were included as fixed effects, and block and paired plot were included as random effects to account for the randomized block design and paired mesocosm structure. This model was specified as: Response ~ Drought * N addition * Spider treatment + (1|Block) + (1|Plot). Before model fitting, the distributions of response variables were examined. Model residuals were inspected using diagnostic plots, and no severe deviations from the assumptions of linear mixed‐effects models were detected.

Here, MBC and MBN represent changes in microbial biomass carbon and microbial biomass nitrogen, respectively. Diversity of soil fauna and microbial communities was estimated using the Shannon‐Wiener diversity index, calculated as: $H^{\prime }=-\sum_{i=1}^{S}\mathrm{pi}\;\ln\mathrm{pi}$, where *H′* is the Shannon diversity index, *S* is the total number of species, and *p*_*i*_ is the proportion of individuals belonging to the *i‐*th species (relative abundance). We calculated the litter decomposition rate for the period during which the spider treatments were imposed using Olson's formula (Olson 1963): *X*_*t*_ = *X*_0_*e ^*−kt*^, where *X*_*t*_ is the mass remaining at time *t*, *X*_*0*_ is the initial mass at *t* = 0, and *k* is the litter decomposition rate content. The results of the linear mixed models were followed by multiple comparisons using the *emmeans* function, and *p*‐values were adjusted using the “Benjamini‐Hochberg” method.

The effects of drought, N addition, spider presence, and their interactions on the community composition of Collembola, other soil fauna, fungi, and bacteria were tested using factorial permutational multivariate analysis of variance (PERMANOVA), with permutations constrained within blocks to account for the randomized block design. Pairwise comparisons with Benjamini–Hochberg adjustment were used to further interpret significant PERMANOVA results. To characterize the shifts in community composition, we performed non‐metric multidimensional scaling (NMDS) using the *vegan* package in *R* based on Bray–Curtis dissimilarity matrices. We used the first two NMDS axes (NMDS1 and NMDS2) as summary variables representing major gradients in community composition for Collembola, other soil fauna, fungi, and bacteria. We further fitted separate LMMs to examine associations between soil biotic metrics and soil N availability or litter decomposition rates. Soil biotic community metrics included abundance, diversity, and NMDS scores for soil fauna; MBC and MBN; and diversity and NMDS scores for fungi and bacteria. Block was included as a random effect.

All statistical analyses were performed in *R* (version 4.5.1) using the packages *emmeans*, *ggplot2*, *lme4*, *lmerTest*, *MuMIn*, *nlme*, and *vegan*. The *R* code for two LMMs is provided in the Supporting Information.

## 3 Results

**FIGURE 1:**
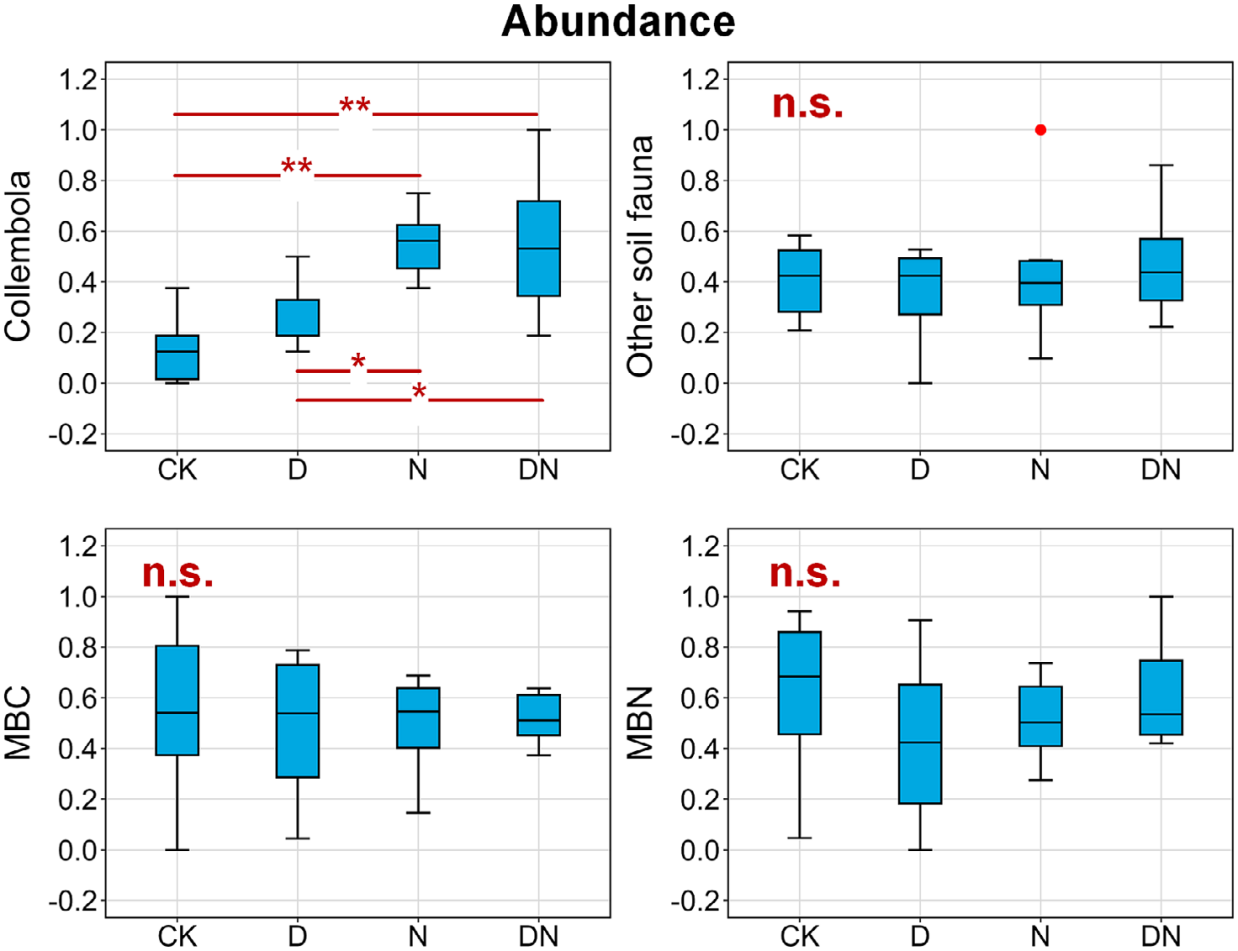
Effects of drought and N addition on normalized spider‐mediated regulation of the abundance of Collembola and other soil fauna, MBC and MBN. Spider‐mediated regulation was calculated as the within‐pair difference between spider‐present and spider‐absent, and these differences were then min‐max normalized to a 0–1 range to facilitate comparisons across variables measured on different scales. In each boxplot, the center line represents the median, the box represents the interquartile range (IQR), and whiskers extend to the most extreme values within 1.5 × IQR. Red points represent outliers beyond 1.5 × IQR. Asterisks denote significant levels: n.s., *p* > 0.05; *p < 0.05; **p < 0.01, respectively.

**FIGURE 2:**
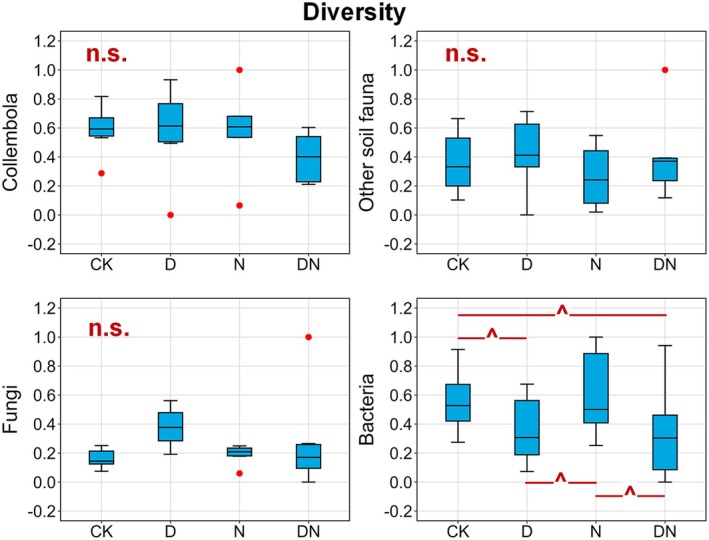
Effects of drought and N addition on normalized spider‐mediated regulation of the Shannon‐wiener diversity of Collembola, other soil fauna, fungi and bacteria. Spider‐mediated regulation was calculated as the within‐pair difference between spider‐present and spider‐absent, and these differences were then min‐max normalized to a 0–1 range to facilitate comparisons across variables measured on different scales. In each boxplot, the center line represents the median, the box represents the interquartile range (IQR), and whiskers extend to the most extreme values within 1.5 × IQR. Red points represent outliers beyond 1.5 × IQR. Statistical notation: n.s., *p >* 0.1; ^0.05 < *p* < 0.1, respectively.

Overall, we found that drought and N addition modulated the relative magnitude of spider‐mediated responses in detrital food webs. The spider‐mediated regulation of Collembola abundance was significantly affected by N addition, but not by drought or their interaction (Table 1), with pairwise comparisons showing that N addition altered this response under both ambient and drought conditions (Figure 1). In contrast, Collembola diversity showed no significant response to spider presence under any treatment (Table 1 and Figure 2). For other soil fauna, neither abundance nor diversity showed significant responses to wolf spider presence across all treatments (Table 1; Figures 1 and 2), suggesting that wolf spider effects were more evident for Collembola than for other soil fauna. For fungal and bacterial communities, spider‐mediated regulation of diversity was mainly affected by drought rather than N addition (Table 1 and Figure 2). Drought significantly affected the spider‐mediated response of bacterial diversity and marginally affected that of fungal diversity, whereas N addition and its interaction with drought did not significantly influence them (Table 1). Pairwise comparisons indicated marginal differences in the spider‐mediated response of bacterial diversity between drought and corresponding non‐drought treatments, either with or without N addition (Figure 2). These results indicate that drought and N addition altered the relative magnitude of spider‐mediated responses, whereas mesocosm‐level models showed no significant effects of spider presence on the response of these variables across treatments (Figures S2 and S3).

**TABLE 1:** Results (*F* values) of the linear mixed models for the effects of drought (D), N addition (N), and their interactions on spider‐mediated regulation of the abundance/biomass and diversity of Collembola, other soil fauna, and microbial communities (fungi and bacteria), soil N availability, and litter decomposition rates.

*F* value	D	N	D:N
Abundance/Biomass			
Collembola	0.6	20.0***↑	0.6
Other soil fauna	< 0.1	0.9	0.2
MBC	< 0.1	< 0.1	0.2
MBN	< 0.1	0.2	1.6
Diversity			
Collembola	0.9	0.8	0.7
Other soil fauna	1.0	0.3	0.2
Fungi	3.5^↑	0.2	0.6
Bacteria	11.0**↓	0.1	0.2
Soil inorganic N content	3.0	0.2	0.2
Litter decomposition rate	1.0	< 0.1	0.5

*Note:* Arrows indicate the direction of significant or marginal treatment effects on spider‐mediated regulation: ↑ indicates an increase and ↓ indicates a decrease in the normalized spider‐mediated response. Asterisks denote significance levels: ^0.05 < *p* < 0.1; **p < 0.01; and ***p < 0.001, respectively.

**FIGURE 3:**
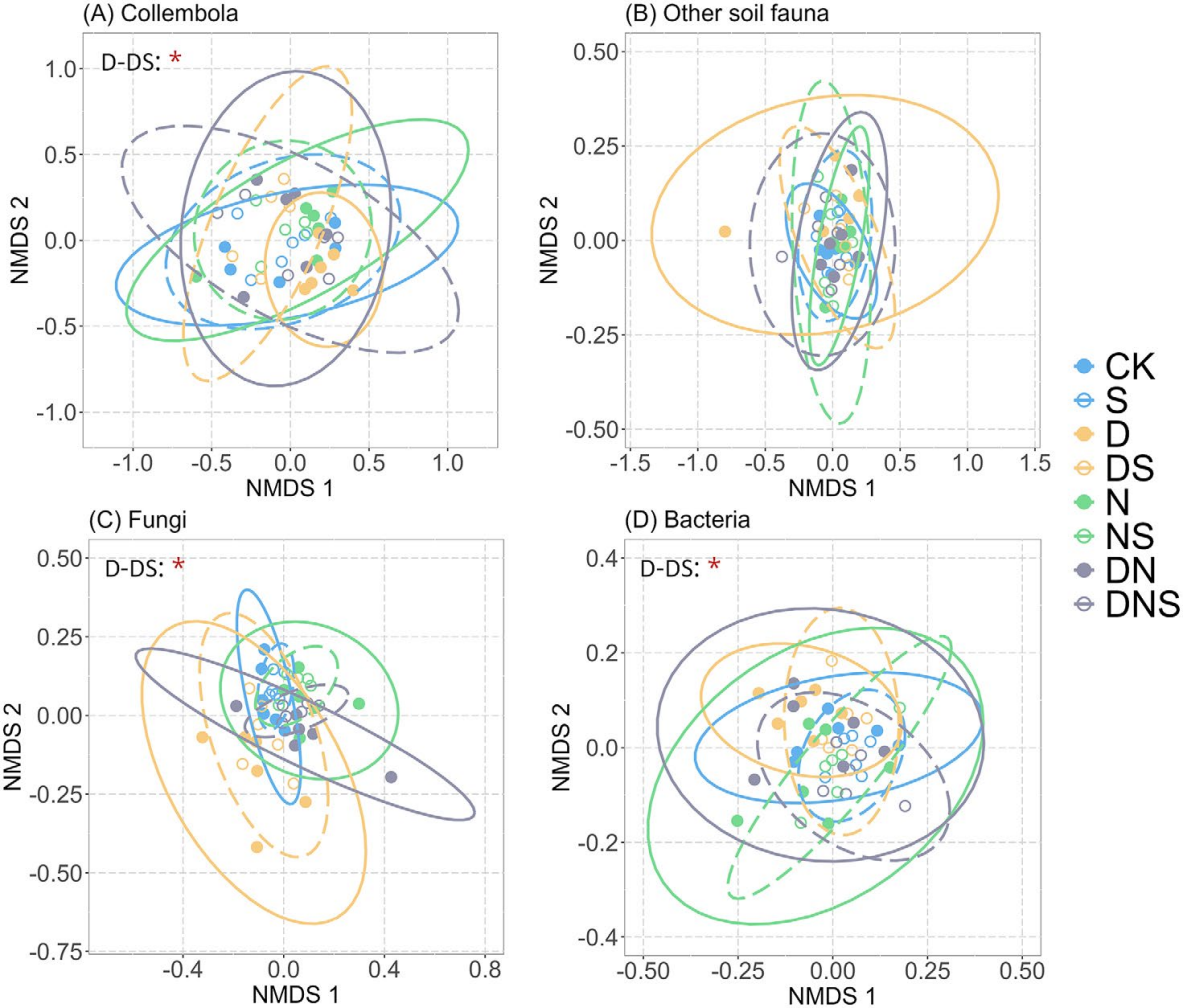
Non‐metric multidimensional scaling (NMDS) ordination plots based on Bray‐Curtis dissimilarities for Collembola, other soil fauna, fungi and bacteria under drought (D), N addition (N), spider presence (S) and their combined treatments. Ellipses indicate 95% confidence regions for each treatment group. Solid ellipses represent spider‐absent treatments, whereas dashed ellipses represent spider‐present treatments. Asterisks indicate significant pairwise PERMANOVA differences between spider‐present and spider‐absent within each drought and N addition treatment combination: *p < 0.05. Detailed PERMANOVA results are shown in Tables S1 and S2.

The results of PERMANOVA showed that Collembola community composition was marginally affected by spider treatment, whereas the composition of other soil fauna was not significantly affected by any treatment or interaction (Table S1). In contrast, the community compositions of fungi and bacteria were significantly affected by drought, N addition, spider treatment, and the interaction of drought and N addition, while other interaction terms were not significant (Table S1). Notably, pairwise comparisons showed significant differences in Collembola, fungal, and bacterial community composition between treatments with and without spider presence under drought conditions, whereas such differences were not detected under ambient or N addition treatments (Figure 3 and Table S2).

**FIGURE 4:**
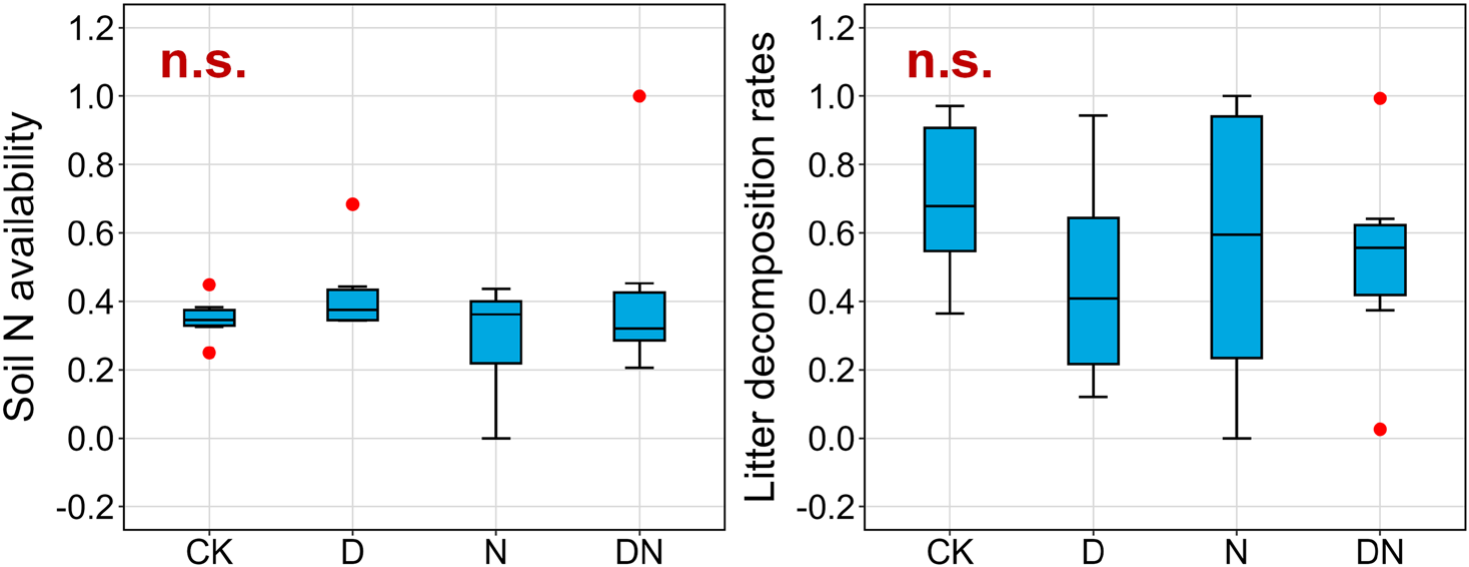
Effects of drought and N addition on normalized spider‐mediated regulation of the soil inorganic N and litter decomposition rates. Spider‐mediated regulation was calculated as the within‐pair difference between spider‐present and spider‐absent, and these differences were then min‐max normalized to a 0–1 range to facilitate comparisons across variables measured on different scales. In each boxplot, the center line represents the median, the box represents the interquartile range (IQR), and whiskers extend to the most extreme values within 1.5 × IQR. Red points represent outliers beyond 1.5 × IQR. Statistical notation: n.s., *p* > 0.1.

**FIGURE 5:**
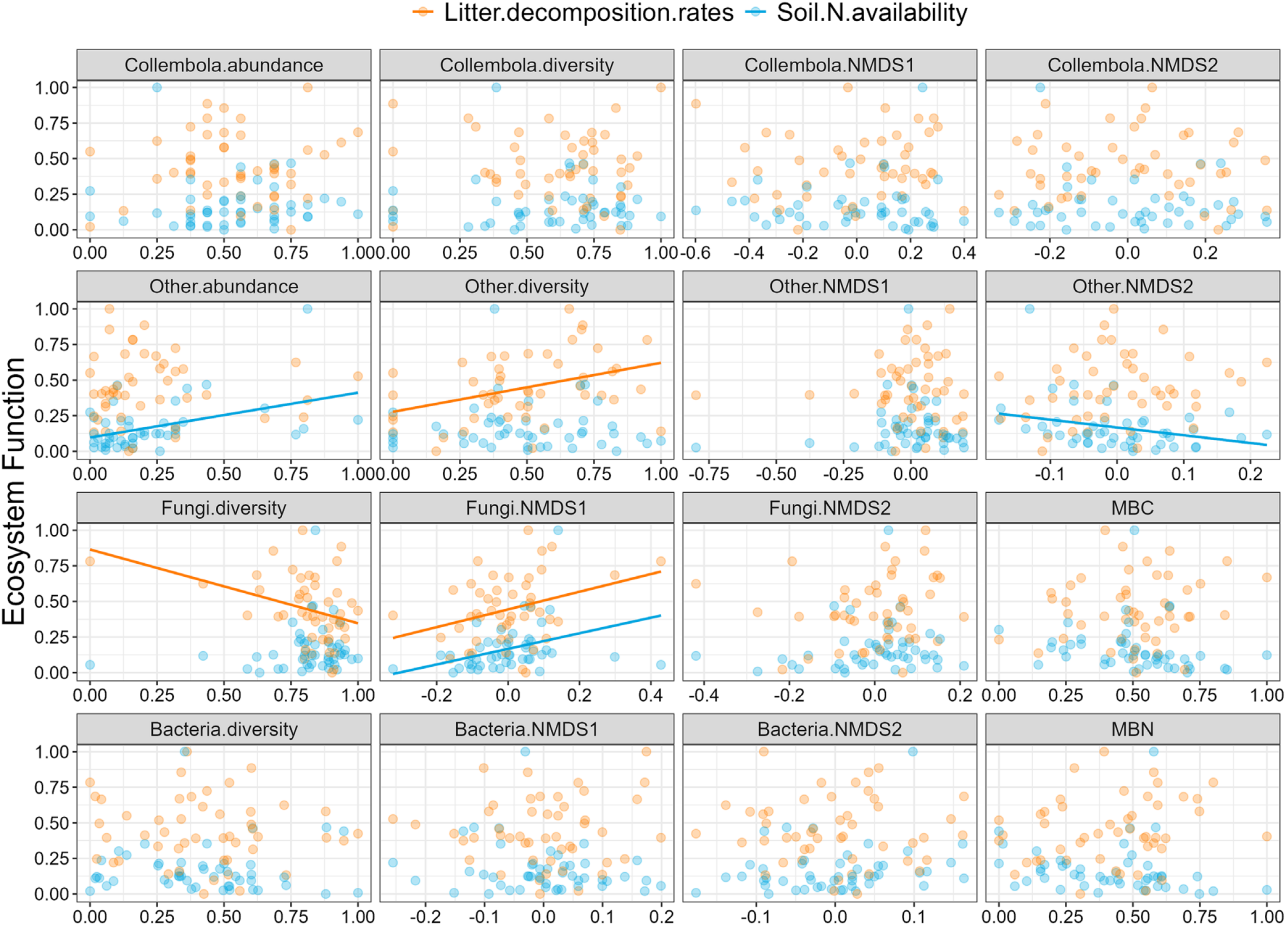
Associations of soil fauna and microbial community metrics with soil N availability and litter decomposition rates. Predictor variables included the abundance, diversity, NMDS1, and NMDS2 of Collembola and other soil fauna; microbial biomass carbon (MBC), microbial biomass nitrogen (MBN); and the diversity, NMDS1, and NMDS2 of fungi and bacteria. NMDS axes summarize major gradients in community composition. Abundance, biomass, and diversity metrics were min–max normalized to a 0–1 range before analysis. Regression lines are shown only for significant relationships based on linear mixed‐effects models (*p* < 0.05).

Across all treatments, soil N availability and litter decomposition rates did not show significant responses to wolf spider presence (Table 1; Figures 4 and S4). However, fungal diversity and community composition were associated with both soil N availability and litter decomposition rates (Figures 4 and 5). In addition, the abundance, diversity, and community composition of other soil fauna were also significantly associated with soil N availability and litter decomposition rates (Figure 5). Although other soil fauna did not respond directly to spider presence (Figures 1 and 2), they were significantly associated with soil N availability and litter decomposition rates (Figure 5).

## 4 Discussion

Interactions among wolf spiders, Collembola, and microbial communities provide insights for understanding how drought and N addition regulate detrital food web dynamics in grassland ecosystems (Bardgett et al. 1998; Lensing and Wise 2006). In this study, both drought and N addition modified the responses of soil fauna and microbial communities to wolf spider presence (Figures 1, 2, 3), indicating that soil moisture and nutrient conditions mediate the strength of top‐down control in detrital food webs (Aupic‐Samain et al. 2021; Liu et al. 2018). Although wolf spider presence did not significantly alter soil N availability or litter decomposition rates (Figures 4 and S4), our findings suggest that wolf spiders reconfigured some components of the detrital food web without producing detectable changes in the ecosystem function variables measured in this study (Figure 5).

Drought altered wolf spider‐associated trophic cascades, leading to shifts in the community composition of Collembola, fungi, and bacteria (Table S2 and Figure 3), as well as changes in fungal and bacterial diversity (Table 1 and Figure 2). Drought reduced Collembola abundance, thereby potentially diminishing their capacity to buffer predator pressure and disrupting the balance of predator–prey dynamics (Figure 1 and Table S3). However, drought did not alter the responses of abundance or diversity of Collembola to wolf spider presence. Furthermore, we found that Collembola community composition was altered by wolf spiders under drought conditions, and that species turnover may have helped maintain overall Collembola abundance (Figure 3). This pattern is consistent with previous studies demonstrating that predator suppression of preferred prey species can be offset by compensatory increases in less preferred prey species (Liu et al. 2016; Koltz et al. 2018), and further suggests that moisture limitation altered the activity of specific Collembola taxa, thereby modifying their interaction strength with predators (Liu et al. 2015; Koltz et al. 2024; Melguizo‐Ruiz et al. 2020). This context dependency may also be partly related to the active hunting strategy of wolf spiders. As ground‐dwelling active hunters, wolf spiders rely on movement and direct encounters with prey across soil and litter habitats. Their top‐down effects therefore depend not only on prey abundance, but also on prey activity, microhabitat use, and soil moisture conditions that influence predator–prey encounter rates. Under drought, reduced soil moisture may constrain the activity of both wolf spiders and Collembola, while also shifting prey distribution toward more moisture‐retaining microhabitats. These changes may weaken spider effects on Collembola abundance while still altering their effects on Collembola community composition.

Such drought‐induced shifts in prey community structure may be linked to changes in microbial communities (Figure S5; Caravaca and Ruess 2014; Koltz et al. 2018). Indeed, spider effects on fungal and bacterial diversity were evident under drought conditions (Table S2), potentially indicating that abiotic stress enhanced the responsiveness of microbial communities to variation in Collembola community composition. Additionally, both fungal diversity and community composition were associated with soil N availability and litter decomposition rates (Figure 5), consistent with the important role of fungi in decomposition and nutrient cycling in grasslands (Lienau et al. 2025). This association may reflect the central role of fungi in decomposing plant litter and cycling nutrients, supported by hyphal growth and the production of extracellular enzymes that break down complex organic compounds. In contrast, bacterial diversity may be less directly related to litter mass loss because bacterial contributions to decomposition often depend on substrate quality, nutrient availability, and local moisture conditions. These associations suggest that fungal communities may be an important microbial link between detrital food web changes and litter decomposition.

Although wolf spider presence did not significantly affect soil N availability and litter decomposition rates (Figure 4), our results indicate that predator‐associated changes in soil fauna and microbial communities did not necessarily cascade to relevant ecosystem functions. One possible explanation is functional redundancy within detritivore and microbial assemblages, which can stabilize ecosystem functioning when top‐down effects alter particular components of the detrital food web (Bardgett and van der Putten 2014; Wagg et al. 2014). In detrital food webs, diverse soil fauna and microbial taxa can perform overlapping functions in litter fragmentation, microbial grazing, organic matter decomposition, and nutrient cycling (Bardgett and van der Putten 2014). Moreover, many detritivores exhibit flexible feeding strategies, which may allow compensatory responses among taxa when predator effects suppress or release specific groups (Coulibaly et al. 2019). This functional overlap and trophic flexibility could buffer soil N availability and litter decomposition against changes in wolf spider‐mediated food web structure (Wagg et al. 2014; Watzinger et al. 2023; Sauma‐Sánchez et al. 2024). In contrast to drought, N addition enhanced the responses of Collembola abundance to wolf spider presence (Table 1 and Figure 1), indicating greater variability in predator–prey dynamics under nutrient‐enriched conditions. Generally, the effects of N addition on Collembola abundance depend on the application rate, with low levels (5–10 g N m^−2^ year^−1^) exerting neutral effects (Chang et al. 2019; Sun et al. 2020; Xu et al. 2007; Eisenhauer et al. 2012; Hu et al. 2022). Nevertheless, even minor soil acidification induced by N addition can significantly increase the sensitivity of Collembola to environmental disturbances, affecting their growth, development, and longevity (Xu et al. 2006, 2009; Hu et al. 2024; Salmon et al. 2002). Notably, although N addition significantly affected Collembola abundance (Table S3), their response to wolf spiders, as indicated by the difference in abundance between spider‐present and spider‐absent mesocosms, remained relatively small. Thus, Collembola responses under N addition appear to be driven more strongly by nutrient‐mediated changes than by top‐down pressure exerted by predators.

Based on the above findings, we inferred that drought and N addition acted largely independently in modulating the relative magnitude of spider‐mediated responses in the detrital food web (Table 1). This pattern may be explained by several mechanisms. First, drought and N addition may impose distinct pressures on soil biota through ecological pathways. Drought primarily imposes stress through water limitation and altered microclimatic conditions, which can reduce organismal activity, mobility, and predator–prey encounter rates (Liu et al. 2015; Siebert et al. 2019). In contrast, N addition acts mainly through resource enrichment by altering nutrient availability, microbial communities, and detritivore resources, potentially favoring taxa with faster growth or higher nutrient demand (Siebert et al. 2019; Fierer et al. 2012). Because these drivers influence detrital food webs through different pathways, their combined effects may not necessarily produce strong synergistic responses (Eisenhauer et al. 2012; Rillig et al. 2019; Wang et al. 2021). Second, functional redundancy within detritivore and microbial communities may buffer the combined effects of drought and N addition. Although the community composition of fungi and bacteria was significantly affected by the interaction between drought and N addition (Table S1), these compositional shifts did not cascade into detectable changes in soil N availability or litter decomposition (Tables 1 and S3). Functional redundancy may occur within detritivore and microbial communities, with different soil fauna taxa potentially contributing overlapping roles in litter fragmentation and microbial grazing, and different microbial taxa potentially contributing overlapping roles in organic matter decomposition and nutrient cycling. Such redundancy, if present, could allow compensatory responses among taxa when some groups are influenced by drought or N addition, thereby weakening functional responses at the ecosystem level (Oliver et al. 2015). However, because we did not directly measure functional traits, feeding guilds, body size, or microbial functional genes, functional redundancy remains a plausible but untested mechanism in this study. Finally, drought and N addition may generate partially opposing effects that reduce the detectable net interaction. Drought may suppress soil biota activity and trophic interactions, whereas N addition may alter nutrient availability and resource conditions, leading to taxon‐specific responses. Because water availability can constrain microbial responses to N addition and water–N interactions can vary with precipitation context, their combined effects may be weak, context dependent, or even partially offsetting (Sun et al. 2015; Rillig et al. 2019). Together, these distinct and potentially offsetting pathways may help explain why drought and N addition did not produce strong interactive effects on most spider‐mediated responses in the detrital food web.

Our results further showed that the other soil fauna groups generally responded weakly to wolf spider presence, whereas Collembola showed clearer responses (Table S4). So, we kept these taxa pooled as other soil fauna to provide a general comparison with Collembola. However, these taxa differ in feeding habits, mobility, and vulnerability to spider predation, and pooling them may have hidden some subtle or contrasting responses. Thus, the nonsignificant response of other soil fauna should be interpreted as a conservative assemblage‐level pattern, rather than evidence that all non‐Collembola taxa were insensitive to spider presence or environmental treatments.

Overall, this study demonstrates that drought and N addition independently regulate top‐down effects of wolf spiders on detrital food webs through distinct impacts, for example, moisture limitation and nutrient enrichment, respectively. Drought altered trophic cascades by altering prey community composition and increasing the sensitivity of microbial communities to predator‐mediated changes in detritivore communities, whereas N addition primarily increased the variability of Collembola abundance responses to wolf spider presence, likely driven by enhanced N availability in detrital food webs. These findings highlight the importance of multi‐trophic interactions and functional redundancy within detrital food webs in maintaining ecosystem functioning under multi‐factorial global change. By integrating belowground biotic interactions with abiotic drivers, our study shows how global change factors can reorganize components of detrital food webs through predator‐associated pathways, with potential implications for understanding belowground ecosystem responses to global change.

## Author Contributions

**Bingchuan Zhang:** conceptualization (equal), data curation (equal), investigation (equal), visualization (equal), writing – original draft (equal), writing – review and editing (equal). **Zhiwei Zhang:** investigation (equal), writing – review and editing (equal). **Wang Ma:** investigation (equal), writing – review and editing (equal). **Zhengwen Wang:** conceptualization (equal), resources (equal), writing – original draft (equal), writing – review and editing (equal).

## Funding

This work was supported by China Postdoctoral Science Foundation (2024M753409), National Natural Science Foundation of China (42230515).

## Conflicts of Interest

The authors declare no conflicts of interest.

## Supporting information


**Table S1:** Results of factorial PERMANOVA testing the effects of drought (D), N addition (N), spider treatment (S), and their interactions on the community composition of Collembola, Other soil fauna, fungi, and bacteria. PERMANOVA was based on Bray–Curtis dissimilarity matrices, with permutations constrained within blocks. Asterisks denote significance levels: ^*p* < 0.1; *p < 0.05; **p < 0.01; and ***p < 0.001, respectively.
**Table S2:** Pairwise PERMANOVA comparisons among treatment combinations for the community composition of Collembola, Other soil fauna, fungi, and bacteria. These analyses were conducted as follow‐up comparisons to the factorial PERMANOVA. *p*‐values were adjusted using the Benjamini–Hochberg method.
**Table S3:** Results (*F* values) of the linear mixed models for the effects of drought (D), N addition (N), and spider (S) and their interactions on the abundance/biomass and diversity of Collembola, other soil fauna and microbial (fungi and bacteria) communities, and litter decomposition rate and soil inorganic N. Models included block and paired plot as random effects to account for the randomized block design and the paired mesocosm structure. Asterisks denote significance levels: ^*p* < 0.1; *p < 0.05; **p < 0.01; and ***p < 0.001, respectively.
**Table S4:** Results (*F* values) of linear mixed models testing the effects of drought (D), nitrogen addition (N), spider presence (S), and their interactions on the abundance and diversity of other soil fauna at the order level. Asterisks denote significance levels: ^*p* < 0.1; *p < 0.05; **p < 0.01; and ***p < 0.001, respectively.
**Figure S1:** Changes in soil moisture under natural and drought conditions during spider experiment treatments.
**Figure S2:** Effects of drought (D), N addition (N) and spider (S) treatments on the abundance of Collembola, other soil fauna, microbial biomass carbon (MBC) and microbial biomass N (MBN).
**Figure S3:** Effects of drought (D), N addition (N) and spider (S) treatments on the Shannon‐wiener diversity of Collembola, other soil fauna, fungi and bacteria.
**Figure S4:** Effects of drought (D), N addition (N) and Spider (S) on the litter decomposition rate and soil inorganic N.
**Figure S5:** Effects of the community composition of Collembola and Other soil fauna on the community composition of Fungi and Bacteria. All regression lines shown are based on the results of a linear mixed model (*p* < 0.05).

## Data Availability

The data that support the findings of this study are openly available in Figshare at https://doi.org/10.6084/m9.figshare.31073551.
