# Supplementary material for: Drought and Nitrogen Addition Modulate Wolf Spider‐Associated Responses in a Detrital Food Web Without Detectable Functional Cascades
Source: Ecol Evol. 2026 Aug 9;16(8):e74165. doi: 10.1002/ece3.74165 (PMC13454221; doi:10.1002/ece3.74165)
Supplement: Supplementary file 1 [file ECE3-16-e74165-s001.docx]

**Supplemental instrument for**

**“Drought and nitrogen addition modulate wolf spider-associated responses in a detrital food web without detectable functional cascades”**

**Table S1** Results of factorial PERMANOVA testing the effects of drought (D), N addition (N), spider treatment (S), and their interactions on the community composition of Collembola, Other soil fauna, fungi, and bacteria. PERMANOVA was based on Bray–Curtis dissimilarity matrices, with permutations constrained within blocks. Asterisks denote significance levels: ^, *p* < 0.1; *, *p* < 0.05; **, *p* < 0.01; and ***, *p* < 0.001, respectively.

| *F* value | Collembola | Other soil fauna | Fungi | Bacteria |
| --- | --- | --- | --- | --- |
| D | 1.0 | 0.9 | **3.4***** | **3.8***** |
| N | 1.1 | 0.7 | **3.5***** | **2.7***** |
| S | **2.3^** | 0.8 | **2.4***** | **2.8***** |
| D:N | 1.2 | 1.2 | **1.6*** | **2.0**** |
| D:S | 0.7 | 0.8 | 0.8 | 0.8 |
| N:S | 1.5 | 1.0 | 0.7 | 0.4 |
| D:N:S | 1.5 | 0.6 | 0.7 | 0.6 |

**Table S2** Pairwise PERMANOVA comparisons among treatment combinations for the community composition of Collembola, Other soil fauna, fungi, and bacteria. These analyses were conducted as follow-up comparisons to the factorial PERMANOVA. P-values were adjusted using the Benjamini–Hochberg method.

| Treatments | Collembola | | Other soil fauna | | Fungi | | Bacteria | |
| --- | --- | --- | --- | --- | --- | --- | --- | --- |
|  | *R*^2^ | *P* | *R*^2^ | *P* | *R*^2^ | *P* | *R*^2^ | *P* |
| CK/S | 0.11 | 0.65 | 0.05 | 0.96 | 0.11 | 0.18 | 0.07 | 0.72 |
| CK/N | 0.04 | 0.83 | 0.04 | 0.96 | 0.13 | 0.03 | 0.11 | 0.24 |
| CK/NS | 0.13 | 0.65 | 0.06 | 0.96 | 0.17 | 0.01 | 0.14 | 0.05 |
| CK/D | 0.22 | 0.32 | 0.13 | 0.96 | 0.13 | 0.01 | 0.18 | 0.02 |
| CK/DS | 0.24 | 0.32 | 0.13 | 0.96 | 0.14 | 0.01 | 0.17 | 0.02 |
| CK/ND | 0.10 | 0.65 | 0.03 | 0.96 | 0.13 | 0.04 | 0.16 | 0.04 |
| CK/NDS | 0.04 | 0.83 | 0.04 | 0.96 | 0.16 | 0.01 | 0.19 | 0.01 |
| S/N | 0.12 | 0.65 | 0.06 | 0.96 | 0.17 | 0.01 | 0.13 | 0.12 |
| S/NS | 0.07 | 0.83 | 0.08 | 0.96 | 0.14 | 0.01 | 0.11 | 0.24 |
| S/D | 0.31 | 0.04 | 0.11 | 0.96 | 0.22 | 0.01 | 0.26 | 0.01 |
| S/DS | 0.10 | 0.77 | 0.08 | 0.96 | 0.17 | 0.01 | 0.17 | 0.01 |
| S/ND | 0.06 | 0.83 | 0.06 | 0.96 | 0.20 | 0.01 | 0.20 | 0.02 |
| S/NDS | 0.04 | 0.89 | 0.07 | 0.96 | 0.20 | 0.01 | 0.17 | 0.01 |
| N/NS | 0.09 | 0.83 | 0.06 | 0.96 | 0.08 | 0.71 | 0.07 | 0.72 |
| N/D | 0.19 | 0.17 | 0.09 | 0.96 | 0.18 | 0.01 | 0.17 | 0.01 |
| N/DS | 0.19 | 0.42 | 0.04 | 0.96 | 0.18 | 0.01 | 0.17 | 0.02 |
| N/ND | 0.06 | 0.83 | 0.06 | 0.96 | 0.12 | 0.10 | 0.13 | 0.08 |
| N/NDS | 0.00 | 0.96 | 0.06 | 0.96 | 0.12 | 0.05 | 0.15 | 0.03 |
| NS/D | 0.30 | 0.12 | 0.12 | 0.96 | 0.23 | 0.01 | 0.24 | 0.01 |
| NS/DS | 0.06 | 0.83 | 0.11 | 0.96 | 0.20 | 0.01 | 0.15 | 0.03 |
| NS/ND | 0.01 | 0.95 | 0.06 | 0.96 | 0.17 | 0.01 | 0.18 | 0.01 |
| NS/NDS | 0.03 | 0.89 | 0.05 | 0.96 | 0.14 | 0.01 | 0.14 | 0.04 |
| D/DS | 0.35 | 0.04 | 0.11 | 0.96 | 0.12 | 0.04 | 0.16 | 0.02 |
| D/ND | 0.22 | 0.18 | 0.10 | 0.96 | 0.13 | 0.01 | 0.15 | 0.05 |
| D/NDS | 0.15 | 0.65 | 0.08 | 0.96 | 0.19 | 0.01 | 0.26 | 0.01 |
| DS/ND | 0.09 | 0.77 | 0.12 | 0.96 | 0.15 | 0.01 | 0.15 | 0.03 |
| DS/NDS | 0.13 | 0.65 | 0.10 | 0.96 | 0.16 | 0.01 | 0.14 | 0.04 |
| ND/NDS | 0.03 | 0.89 | 0.07 | 0.96 | 0.09 | 0.46 | 0.11 | 0.24 |

**Table S3** Results (*F* values) of the linear mixed models for the effects of drought (D), N addition (N), and spider (S) and their interactions on the abundance/biomass and diversity of Collembola, other soil fauna and microbial (fungi and bacteria) communities, and litter decomposition rate and soil inorganic N. Models included block and paired plot as random effects to account for the randomized block design and the paired mesocosm structure. Asterisks denote significance levels: ^, *p* < 0.1; *, *p* < 0.05; **, *p* < 0.01; and ***, *p* < 0.001, respectively.

| *F* value |  | D | N | S | D:N | D:S | N:S | D:N:S |
| --- | --- | --- | --- | --- | --- | --- | --- | --- |
| Abundance/Biomass | Collembola | **4.6*** | **4.3*** | 1.4 | 1.0 | 0.4 | 0.3 | 0.1 |
|  | Other soil fauna | 0.1 | 1.1 | <0.1 | <0.1 | <0.1 | 0.8 | 0.2 |
|  | MBC | 0.5 | **4.0^** | 2.8 | <0.1 | <0.1 | <0.1 | 0.2 |
|  | MBN | 1.1 | 0.6 | <0.1 | <0.1 | 0.1 | 0.2 | 2.0 |
| Diversity | Collembola | 1.8 | <0.1 | **3.7^** | 0.2 | 1.1 | 1.0 | 0.9 |
|  | Other soil fauna | 0.8 | <0.1 | <0.1 | **3.9^** | 1.2 | 0.4 | 0.2 |
|  | Fungi | **2.7^** | 0.2 | **3.9^** | 0.8 | **3.5^** | 0.2 | 0.6 |
|  | Bacteria | **10.4**** | 1.7 | **9.3**** | 2.8 | **3.8^** | <0.1 | <0.1 |
| Litter decomposition rate | | **4.8*** | 0.8 | 2.2 | 0.2 | 1.1 | <0.1 | 0.6 |
| Soil inorganic N | | <0.1 | **92.**** | 0.8 | 1.0 | 2.1 | 0.1 | 0.2 |

Table S4 Results (*F* values) of linear mixed models testing the effects of drought (D), nitrogen addition (N), spider presence (S), and their interactions on the abundance and diversity of other soil fauna at the order level. Asterisks denote significance levels: ^, *p* < 0.1; *, *p* < 0.05; **, *p* < 0.01; and ***, *p* < 0.001, respectively.

| *F* value |  | D | N | S | D:N | D:S | N:S | D:N:S |
| --- | --- | --- | --- | --- | --- | --- | --- | --- |
| Abundance | Coleoptera | <0.1 | 0.8 | <0.1 | 0.2 | 0.7 | 1.8 | <0.1 |
|  | Hymenoptera | 1.6 | 5.1* | 0.3 | 3.1 | 0.1 | 1.2 | 0.4 |
|  | Other (Diptera larvae, Psocoptera) | <0.1 | 1.1 | 1.4 | 2.8 | 0.1 | 0.7 | 0.5 |
| Diversity | Coleoptera | 0.5 | <0.1 | 1.1 | 1.0 | <0.1 | 2.4 | 0.4 |
|  | Hymenoptera | <0.1 | 0.1 | <0.1 | 3.6^ | 0.1 | 0.9 | <0.1 |
|  | Other (Diptera larvae, Psocoptera) | 0.8 | <0.1 | 0.1 | 3.4^ | <0.1 | 1.1 | 1.0 |

**Figure S1** Changes in soil moisture under natural and drought conditions during spider experiment treatments.

**Figure S2** Effects of drought (D), N addition (N) and spider (S) treatments on the abundance of Collembola, other soil fauna, microbial biomass carbon (MBC) and microbial biomass N (MBN).

**Figure S3** Effects of drought (D), N addition (N) and spider (S) treatments on the Shannon-wiener diversity of Collembola, other soil fauna, fungi and bacteria.

**Figure S4** Effects of drought (D), N addition (N) and Spider (S) on the litter decomposition rate and soil inorganic N.

**Figure S5** Effects of the community composition of Collembola and Other soil fauna on the community composition of Fungi and Bacteria. All regression lines shown are based on the results of a linear mixed model (*p* < 0.05).

**The R code for two linear mixed-effects models**

library(lme4)

library(lmerTest)

library(car)

\## Spider-mediated regulation model for Table 1

\## spider_effect = spider-present value - spider-absent value

mod_spider_effect <- lmer(

spider_effect ~ D * N + (1 | Block), data = dat_spider_effect, REML = TRUE)

Anova(mod_spider_effect, type = 3)

\## Original mesocosm-level model for Table S2

mod_original <- lmer( variable ~ D * N * S + (1 | Block) + (1 | Plot),

data = dat, REML = TRUE)

Anova(mod_original, type = 3)
